# Unusual presentation of thyrotoxicosis as complete heart block and renal failure: a case report

**DOI:** 10.1186/1752-1947-3-9303

**Published:** 2009-11-28

**Authors:** Suresh Krishnamoorthy, Rajay Narain, John Creamer

**Affiliations:** 1 City Hospital, Sandwell and West Birmingham Hospitals, NHS Trust, Birmingham, B18 7QH, UK; 2 University Hospitals of North Staffordshire, Stoke on Trent, ST4 6QG, UK

## Abstract

**Introduction:**

Thyrotoxicosis is a clinical entity often very difficult to diagnose without biochemical confirmation as its clinical features can be highly varied. The most common cardiac manifestations of thyrotoxicosis are resting sinus tachycardia, supraventricular tachycardia including atrial fibrillation and atrial flutter with or without cardiac failure. Bradycardia and atrio-ventricular conduction defects are very uncommon in thyrotoxicosis.

**Case presentation:**

We report the case of a 59-year-old Caucasian man presenting with progressive weight loss, abnormal liver function, acute renal failure and complete heart block due to thyrotoxicosis.

**Conclusion:**

Thyrotoxicosis should be considered as a possible diagnosis in patients with bradycardia and heart blocks associated with abnormal symptoms like weight loss. Nevertheless, the clinical, electrophysiological and biochemical abnormalities associated with thyrotoxicosis may be completely reversible restoring euthyroid state.

## Introduction

Hyperthyroidism (thyroid overactivity, thyrotoxicosis) is more common in women (2% to 5%), with female to male sex ratio of up to 5:1 between the ages of 20 to 40. This multisystem disorder is characterised by hyperthermia, tachycardia and/or palpitations, weight loss, tremor, diarrhoea, loss of libido, eye problems, muscle weakness, restlessness, severe agitation and altered mental status. However the classical features may not be evident in the 'apathetic variant'. Thyroid storm is often life-threatening and even more difficult to diagnose with unusual presenting features such as status epilepticus, cerebral infarction or acute renal failure. We report the case of a 59-year-old Caucasian man admitted with weight loss, lethargy, abnormal liver function, renal failure and complete heart block due to thyrotoxicosis.

## Case presentation

**Figure 1:**
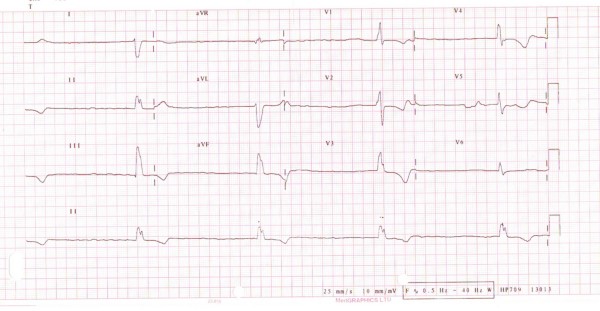
**Patient's electrocardiogram at the time of presentation when thyrotoxic showing the underlying rhythm as atrial fibrillation with complete atrio-ventricular heart block**.

A 59-year-old Caucasian man was admitted with lethargy and progressive weight loss for 6 to 8 months prior to presentation. He had no history of loss of appetite, diarrhoea, excessive sweating, heat intolerance or visual disturbances. His medical history included ankylosing spondylosis with previous prosthetic aortic valve replacement for aortic regurgitation 20 years before presentation. Physical examination revealed an emaciated and lethargic man with a body mass index of 16. He was slightly pale, jaundiced and febrile with a diffused non-tender multinodular goiter with tremor on upper extremities. His pulse rate was 40/minute with a blood pressure of 110/70 mmHg. Examination of his cardiovascular system revealed prosthetic S2 without any evidence of aortic regurgitation. There were no signs of heart failure or infective endocarditis. His lungs were clear and his abdomen revealed two-finger breath tender hepatomegaly with smooth edges without any ascites or splenomegaly. Investigations revealed the following: haemoglobin 9.8 gm/dl, mean cell volume 88.6 fl, white cells 5.2 × 10^9^/L, platelets 80 × 10^9^/L, potassium 5.6 mmol/L, urea 18 mmol/L, creatinine 250 μmol/L, blood glucose 8.9 mmol/L, C-reative protein 66 mg/L, magnesium 0.94 mmol/L, bilirubin 54 μmol/L, alkaline phosphatase 187 μ/L, alanine aminotransferase 80 μ/L, gamma glutamyl transferase 184 μ/L, total protein 82 gm/L, albumin 34 gm/L and adjusted calcium 2.77 mmol/L. On top three sets of blood cultures were negative of bacterial aerobic and anaerobic growths. His chest X-ray showed cardiomegaly but no pulmonary oedema. However his 12-lead electrocardiogram showed complete heart block with underlying atrial fibrillation (Figure 1). Interestingly his thyroid function confirmed a toxic state with thyroid stimulating hormone of < 0.03 mU/L, tri-iodothyronine (T3) at 24.1 pmol/L, and free thyroxine (T4) at > 100 pmol/L. Of note, the autoimmune thyroid antibodies were negative for both thyrotropin receptors and microsomes. A transthoracic echocardiogram revealed stable aortic valve prosthesis without any regurgitation. His left ventricle was notably dilated but did have a preserved good systolic function and no other valvular abnormalities were seen. However the ultrasonogram (USG) of his abdomen revealed a smoothly enlarged liver without any evidence of focal lesions or biliary obstruction. Similarly USG his neck confirmed enlarged thyroid gland with multiple nodules.

**Figure 2:**
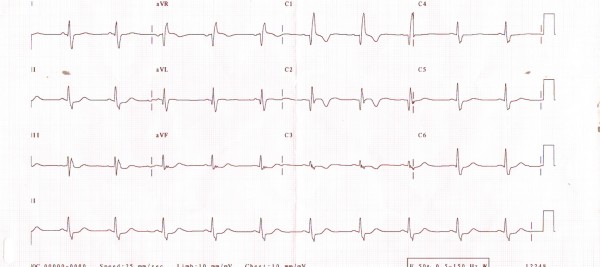
**Patient's electrocardiogram after restoring euthyroid state with carbimazole showing sinus rhythm with normalisation of atrioventricular heart block**.

The patient was briefly managed with temporary pacing during the period of the hemodynamic instability. He was treated with 40 mg of carbimazole once daily for 3 weeks, subsequently achieving both clinical and biochemical euthyroid state with improvement in his renal functions. Similarly his heart rhythm was stabilised restoring sinus rhythm with PR interval of 180 msec with normal QRS complexes (Figure 2). His subsequent prolonged cardiac monitoring showed sinus rhythm without any evidence of bradycardia or heart blocks. Subsequently, the patient underwent an uncomplicated subtotal thyroidectomy.

## Discussion

Documentation related to thyrotoxicosis show a variety of cardiac arrhythmias [1], usually sinus tachycardia (40%) and atrial fibrillation (10 to 22%) with or without heart failure due to the intrinsic effects of thyroxine on sinoatrial (SA) node electrophysiological function[2]. Although tachyarrhythmias are common, atrioventricular (AV) conduction defects and SA blocks have been reported in patients with hyperthyroidism [3]. Second and third degree heart blocks are very rare. Over the last four decades, only a few cases of complete heart block complicating thyrotoxicosis among patients have been reported [4,5]. Factors contributing to the development of heart block in these cases are administration of rate-controlling drugs, abnormal electrolytes and the presence of valvular heart disease [6]. Lack of awareness of the above association and atypical presentations may delay diagnosis and treatment. A patient's hypercatabolic state leads to weight loss, while liver congestion or hepatocellular dysfunction causes abnormal liver functions. Meanwhile, renal failure may be a manifestation of low cardiac output state during the period of bradycardia.

The exact cause of AV conduction abnormalities is unknown, but the majority of cases are preceded by acute infections. In one such case, post mortem examination revealed that polymorphs and gram-positive cocci permeated the AV node. However focal myocarditis around the AV node and interstitial inflammation of the AV node and bundle has also been reported in autopsies [7]. On top, T3 may also have an effect on the myocardium and on the patient's electrophysiological function. Indeed autopsies in patients with hyperthyroidism showed dilated ventricles, myocyte hypertrophy, myocyte necrosis, myocardial oedema, interstitial and perivascular fibrosis [8].

Only a few reported cases show complete to partial reversal of conduction abnormalities [9] and heart failure [10] withjavascript:PopUpMenu2_Set(Menu9919355); correction of thyroid status during the course of treatment. Therefore, cardiac and electrophysiological decompensation due to thyrotoxicosis may be completely reversible.

## Conclusion

AV blocks, especially complete heart block, rarely occur in thyrotoxicosis. Clinical status and conduction abnormalities are probably reversible when the euthyroid state is restored. There should be a high index of clinical suspicion in considering a thyrotoxic state when cachexia is and cachexia should be associated with heart rhythm abnormalities, particularly heart blocks. The administration of rate controlling drugs (beta blockers, calcium channel blockers) often worsens the condition of heart blocks, which can be life-threatening. Patients who have a long-standing and unstable severe disease are at high risk. We present the first reported case of a patient with thyrotoxicosis presenting with unusual features of weight loss, abnormal liver function tests and acute renal failure with complete heart block whose conduction and biochemical abnormalities were completely resolved after restoring his euthyroid state.

## Abbreviations

AV: atrioventricular; SA: sinoatrial node; T4: thyroxine; T3: tri-iodothyronine.

## Consent

Written informed consent was obtained from the patient for publication of this case report and any accompanying images. A copy of the written consent is available for review by the Editor-in-Chief of this journal.

## Competing interests

The authors declare that they have no competing interests.

## Authors' contributions

SK contributed in writing the manuscript and the performed literature search on the topic. RN and JC were involved in patient care. JC also supervised the writing of the manuscript. All authors read and approved the final manuscript.
